# Comparison between velocity‐specific exercise and isometric exercise on neck muscle functions and performance: a randomised clinical trial

**DOI:** 10.1186/s12891-021-03943-0

**Published:** 2021-01-14

**Authors:** Sharon MH Tsang, Kenneth TK Chan, Parco LT Ho, Jacky CY Kwok, Daniel HT Tse, Henry HT Tsoi

**Affiliations:** Department of Rehabilitation Sciences, The Hong Kong Polytechnic University, Yuk Choi Road, Hung Hom, Kowloon, Hong Kong, China

## Abstract

**Background:**

Evidence has shown that velocity-specific exercise results in additional benefits for peripheral joint muscles by promoting their functions, however, its effects on spinal muscles are yet to be investigated. This study aimed to examine the feasibility and effects of velocity-specific exercise compared to isometric exercise on cervical muscle functions and performance in healthy individuals.

**Methods:**

Thirty healthy adults were randomised to practise either the velocity-specific exercise (VSE, *n* = 15) or isometric exercise (IE, *n* = 15) for 6 weeks. Functions and performance of the cervical extensors and flexors were assessed pre- and post-program, by analyzing the peak torque and electromyography during the isokinetic testing, and cross-sectional area of the deep cervical muscles at rest. The self-reported level of difficulty and post-exercise soreness during the exercise were recorded to evaluate the feasibility and safety of the two exercise programs.

**Results:**

Both VSE and IE exercises resulted in significant improvement of the muscle functions and performance while there were no between-group differences at reassessment of the (a) cross-sectional area of longus colli and semispinalis capitis; (b) EMG amplitude in sternocleidomastoid and cervical erector spinae, and (c) peak torque values. Further analysis revealed that degree of correlation between extension torque and EMG amplitude of cervical erector spinae increased in both groups. However, significant correlation was found only in VSE group post-program. There were no significant differences for the level of difficulty and post-exercise soreness found between two groups.

**Conclusions:**

Both velocity-specific and isometric exercises significantly promoted cervical muscle functions and performance. The present study confirms that velocity-specific exercise can be practised safely and it also contributes to a greater enhancement in neuromuscular efficiency of the cervical extensors. These findings indicate that the velocity-specific exercise can be considered as a safe alternative for training of the cervical muscles. Further study is recommended to examine its benefit and application for promoting the muscle functions and recovery in symptomatic individuals.

## Background

Nonspecific neck pain is the fourth leading cause of chronic disability, with an annual prevalence rate higher than 30% [1]. It was reported that as high as 50% of the patients continue to experience some degree of neck pain in one-year period [1, 2]. Individuals with neck pain showed various forms of neuromuscular dysfunctions in their neck muscles, in which the manifestation of over-activity of the superficial cervical flexors, namely the sternocleidomastoid and anterior scalene even at low load task execution is most prevalent [3–5]. Such motor reorganisation pattern remains as one of the classical motor control strategies for compensation of the underlying neuromuscular deficiencies of the deep layer of cervical muscles [3, 6, 7]. The neuromuscular deficiencies include weakness, inhibition and delayed activation of longus colli and longus capitis. Due to the practical limitation in accessing the deep layer of cervical muscles non-invasively, studying the activity level of the superficial cervical flexors is considered to be the best alternative for evaluation of the neuromuscular deficiencies in neck pain research [3, 8–10]. These neuromuscular deficiencies have been suggested to be the result of impairment of the underlying motor control and postural stability of the cervical spines [3, 7]. It was advocated that restoring muscle endurance and optimising the cervical muscle synergy help relieving neck pain and promoting recovery [11, 12].

Training the deep layer of the cervical muscles (i.e. longus colli, multifidus and semispinalis capitis and cervicis) and optimising the neuromuscular efficiency may therefore help promote rehabilitation outcomes of neck pain. Evidence from a systemic review indicates that exercise has medium level and significant pain reduction effects in both short term (Hedges' g = -0.53) and intermediate term (Hedges' g = -0.45) [13]. Amongst the various types of exercises, deep neck muscle activation exercise (using cranio-cervical flexion), isometric exercises and isotonic exercises of the neck muscles were the most prevalently used in clinical trials. Despite the evidence that advocates the use of velocity-specific exercises for its additional benefits for rehabilitation of the muscles for research in peripheral joints [14, 15], no study has been conducted to investigate the feasibility as well as the effectiveness of using the velocity-specific exercise for the cervical spine.

Exercising or training of muscle at specific movement speed could be a more functional approach to address the mechanical demands associated with the daily activities compared to isometric exercise [16, 17]. Daily activities often involve movements of the neck, therefore isometric work is likely to be less effective to simulate the requirement of motor control of the cervical spine. Furthermore, there may also be additional benefits to improve the cervical muscle functions by enhancing the muscle contractility for the greater level of mechanical stimulus that acts on muscle tendons when exercise is performed at faster speed [15]. Training of velocity-specific and explosive muscle contractions improved torque production at greater range of motion, compared to sustained contractions [18]. Moreover, it was shown that exercise performed at specific pace resulted in greater pain reduction compared to the use of isometric exercise for rehabilitation of knee osteoarthritis [14]. The movement during the exercise promotes substance exchange within the articulation and pain relief. There were some concerns of excessive shearing force produced by neck muscles during velocity-specific exercise performed at high speeds. It was reported that there was high shearing force during high-speed eccentric contraction from isokinetic efforts of knee flexors [19]. With measures such as practising the exercise at adjusted speed level with regard to the respective anatomy and biomechanics of the body part, proper screening to rule out those with joint laxity and practicing the exercise training with accuracy would be most important to ensure the safety of the exercise implementation.

There was no previous research studying the effects of exercise performed at specific velocity on cervical muscles despite the additional benefits reported over isometric exercise found in peripheral joint rehabilitation. Therefore, the present study aimed to investigate whether velocity-specific exercise could be implemented safely in healthy individuals (i.e. feasibility) and if such exercise approach would be better or of similar efficiency to isometric exercise in terms of the neck functions, performance and size of the deep layer of the cervical muscles. The findings of this study help provide insights in research of neck exercises to determine if (a) velocity-specific exercise are safe to perform in the cervical spines and (b) effective to promote neck muscle functions and performance in healthy participants, before proceeding to the next step for exploring its clinical implications in rehabilitation of individuals with neck pain.

## Methods

### Study design

This study was a single-blinded randomized controlled trial comparing the effects of a course of velocity-specific exercises (VSE) and isometric exercises (IE) on the functions and performance of the neck muscles.

### Participants

Thirty adults (21 Males, 9 Females), aged from 19 to 23 who were asymptomatic in their neck region for the last 12 months, were recruited by convenient sampling at University campus. Individuals were excluded if they reported to have any of the following conditions: vestibular or sensory abnormalities; vertebral artery insufficiency; previous trauma or surgery of the brain or spine; known cervical orthopaedic pathology; infectious disease; inflammatory, systemic or neoplastic origin; or any contraindication for performing neck strengthening exercise. Ethics approval was obtained from the Departmental Research Committee of the Hong Kong Polytechnic University, and written informed consent was gained from each participant. This study has obtained the clinical trial registry (Registry number: ChiCTR2000037837) and all assessments were conducted at the Clinical Research Laboratory of The Hong Kong Polytechnic University between June and November 2019.

### Experimental procedures and measurements

**Fig. 1:**
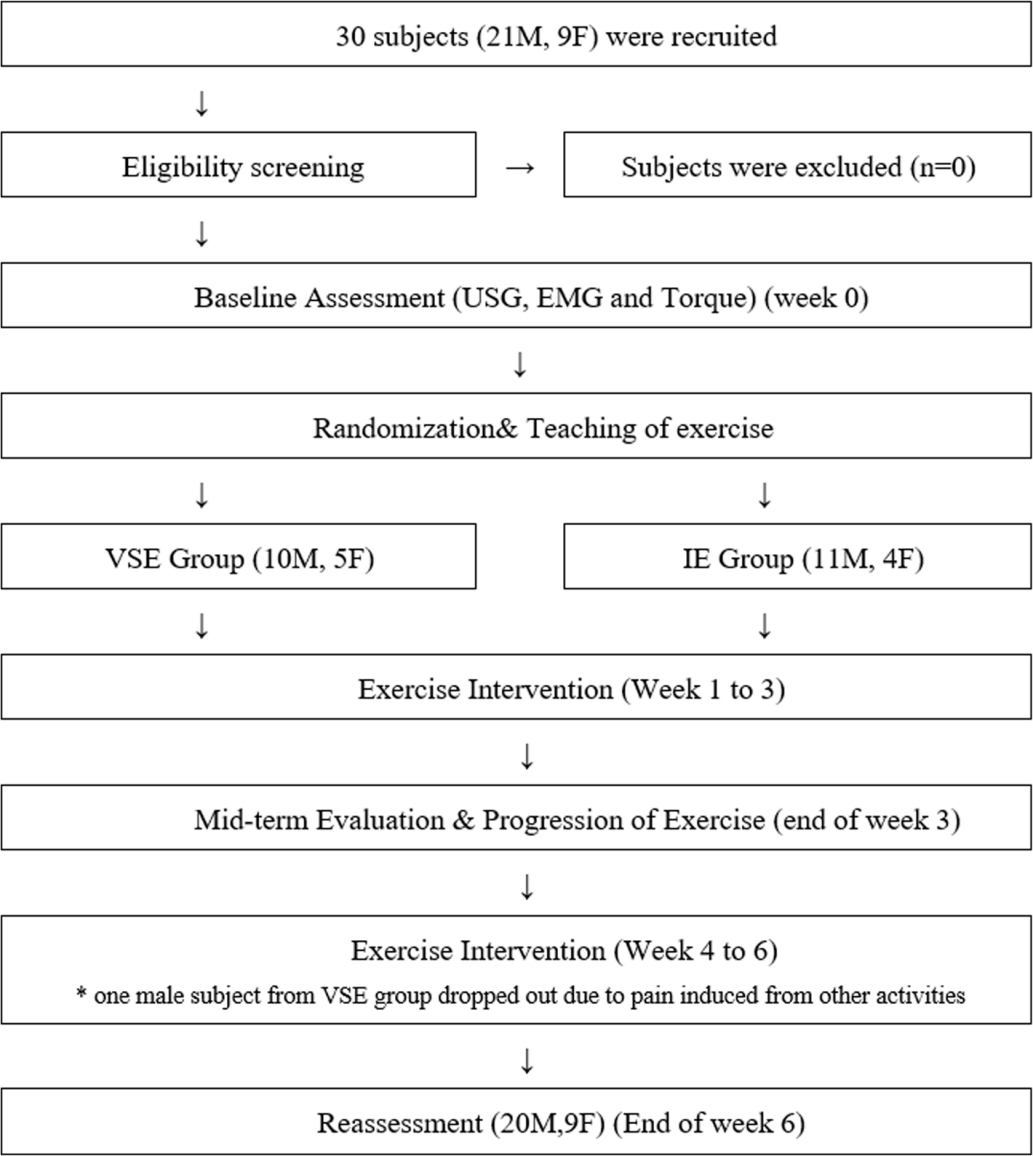
Study design

Figure 1 shows the study design. After completing baseline assessments, participants were randomly allocated to the VSE group or IE group by drawing lots. Participants were blinded to the allocation of the exercise type.

Mid-term follow-ups were conducted after week 3 to ensure movement quality and to progress the neck exercise program. Each participant was prescribed with an elastic band that matched the neck strength of the individual for progression of the exercise program. Reassessments using the same protocol were conducted after week 6. The functions and performance of the neck muscles were assessed by electromyography (EMG) amplitude and torque production during neck flexion and extension phases in isokinetic testing, and the size of the deep neck muscles was assessed by ultrasonography.

#### EMG activity of the neck flexors and extensors during isokinetic assessment

Standardised skin preparation was practised prior to the placement of EMG electrodes, which included hair removal, light abrasion using sandpaper and cleansing of skin using alcohol swab and the electrode-skin impedance was < 10 kΩ. The placement of surface EMG electrodes has been reported by Lascurain-Aguirrebeña et al. [20] and recommended by SENIAM [31]. Surface EMG of sternocleidomastoid and cervical erector spinae were measured at a sampling frequency of 1024 Hz (Noraxon Telemyo Wire system 9000, USA Inc.) using the bipolar disposable Ag–AgCl disc-shaped electrodes of 10 mm diameter with 20 mm inter-electrode distance. For sternocleidomastoid, electrodes were placed adjacent to a point 30% of the distance from the sternal notch to the mastoid process, along the direction of muscle fibres. For cervical erector spinae, electrodes were placed 2 cm lateral to the spinous process of C4-5 spinous process, over the muscle bulk, along the direction of muscle fibres.

For comparison of the effort of the activation between muscles and participants, the EMG amplitude was normalized to the Maximal Voluntary Contraction (MVC) of the respective muscles. MVC value of sternocleidomastoid and cervical erector spinae were recorded 3 times each in supine and prone position respectively using isometric neck contraction against manual resistance at mid-range. The mean of the two peak EMG amplitude during flexion phase and extension phase of the two isokinetic test trials of the neck flexors and extensors was taken and displayed as %MVC. Cervical movements was measured by inertial motion sensor system (MyoMotion, Noraxon, USA Inc.) with two motion sensors being placed over the forehead and C7 spinous process respectively. The cervical kinematics were recorded at a sampling frequency of 100 Hz during the isokinetic assessment for a better comprehension of the relationship between the EMG amplitude and torque.

#### Torque performance of the neck flexors and extensors during isokinetic assessment

**Fig. 2:**
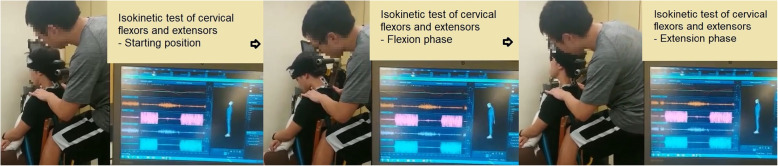
Isokinetic assessment of the neck flexors and extensors at velocity of 30°/second with synchronized recording of 3D cervical spine motion and electromyography

Torque values of the flexion and extension phase were measured using isokinetic assessment system (CYBEX isokinetic dynamometer) (Fig. 2). The participants were securely stabilized on a wooden chair with straps to enable isolated cervical movements from the trunk. The fulcrum of the dynamometer was aligned at the C7 level. The resistance pad was placed at the occiput area with straps across forehead. Head weight of the participants was recorded during calibration to normalize the torque production value by eliminating gravity effects. Range of motion for testing was set individually with an adjustment of 10° short from their end-range to avoid excess load or shearing force acting on the neck during testing. The maximal isokinetic torque was determined at 30°/s angular velocity. Participants were asked to familiarize with the isokinetic testing procedures before the actual test. After the practice, 2 cervical isokinetic test trials with participant moved through their neck extension to flexion range were performed for 5 repetitions, with 1-minute rest between trials. Participants were encouraged to execute the test as hard as they could while maintaining chin tucked-in to avoid excessive neck protraction during the trial. At least 3 peak values from each trial were taken and the mean was obtained as the torque value.

The integrated analysis of the value of torque and EMG amplitude during flexion and extension phase offers the evaluation of changes in the neuromuscular efficiency between the deep and superficial muscles of the neck in response to neck exercise program.

#### Cross‐sectional area of the deep neck flexors and extensors assessment using ultrasonography

**Fig. 3:**
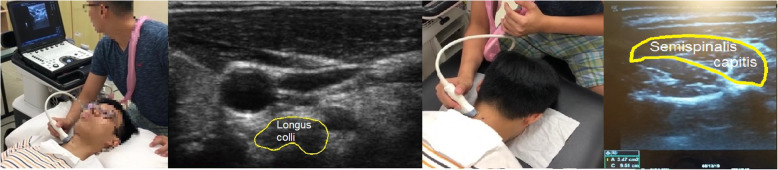
Measurement of the cross-sectional area of longus colli and semispinalis capitis

Ultrasound scanning of the cross-sectional area of deep cervical flexors, i.e. longus colli and deep cervical extensors, i.e. semispinalis capitis were measured by the ultrasound imaging unit (LOGIQe, GE Healthcare) using the B mode application, at a depth of 3–4 cm with the linear transducer frequency set to 8 MHz was used (Fig. 3). Measurement of the cross-sectional area of each muscle was taken twice and the mean was used for analysis. For longus colli, participants were placed in supine with head supported by a pillow. Level C4 in anterior neck was estimated and marked by posterior neck palpation. Bifurcation of carotid artery was found to ensure C4 level was identified. The assessor then moved the probe distally until the bifurcation disappeared and the image of longus colli was taken at that position.

To visualize the semispinalis capitis, participants were placed in prone position and C4 spinous process was marked and transducer was placed lateral to the marking. Semispinalis capitis was identified in the third muscle layer. The bright line represents the fascial layer and the area enclosed by the line refers to the cross-sectional area of semispinalis capitis (Fig. 3) [21].

### Implementation of two exercise programs

#### Velocity‐specific exercise program (VSE)

For VSE group, participants were instructed to perform neck flexion and extension exercise 3 days/week for 6 weeks consecutively. The neck flexion exercise was performed in supine while neck extension exercise was in 4-point kneeling position. Participants were required to maintain the chin tucked-in throughout the flexion exercise. 60 beats per minute (BPM) was selected as the velocity of neck movement during both exercises. The speed was determined by the data collected in our preliminary investigation of the neck movement velocity during common daily activities.

**Fig. 4:**
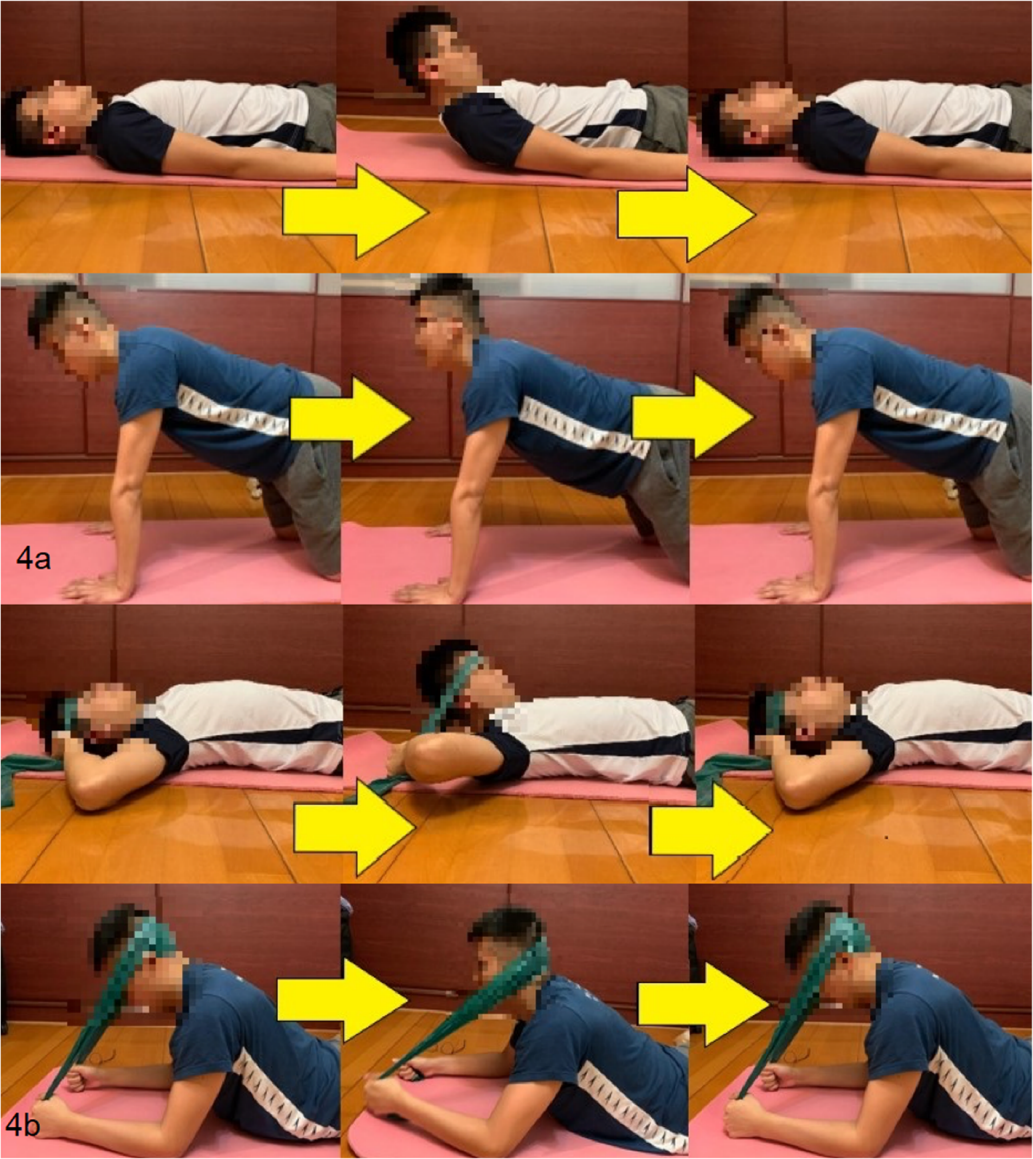
**a **Velocity-specific exercise during week 1-3; and **b** During week 4-6 with progression using elastic band

A metronome set at 60 BPM was used. At the first “beep”, participants started to perform the exercise from neutral position. By the time that their neck reached the end range of either flexion or extension, the participants heard another “beep”. After that, they moved their neck from end range position to neutral position at the exact time of another “beep” (Fig. 4a & b). The above procedures were repeated 6 times as one set for 3 sets, with 1-minute rest between sets.

#### Isometric exercise program (IE)

**Fig. 5:**
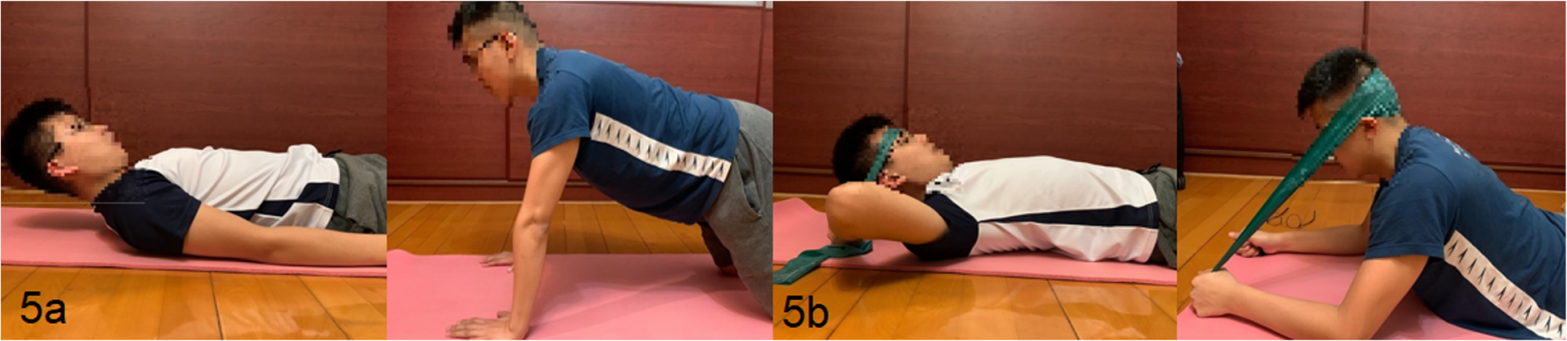
**a** Isometric exercise during week 1-3; and **b** During week 4-6 with progression using elastic band

For IE group, the neck exercise was performed at the same frequency i.e. 3 days/week for 6 weeks consecutively (Fig. 5a & b). Participants were asked to maintain their neck at mid-range position, with chin tucked-in in supine and mid-range position in 4-point kneeling respectively for 12 seconds, repeated for 3 sets, with 1-minute rest between sets.

#### Progression of exercises

Weekly progression of the exercise was made after week 3 according to the performance and responses from the individual. For week 1–3 period, participants were asked to perform the exercise with body weight. After mid-term evaluation, participants were taught to practise the neck exercises using the elastic band (Thera-band). To facilitate a better direction of resistance, the position of the extension exercise had been modified to elbow-prone position. The position of flexion exercise remained unchanged (Figs. 4b and 5b).

A targeted level of exertion was set to standardise the exercise level. During week 4 to 6, the targeted level of exertion was set at 4/10 to 6/10 in the Borg scale respectively, with 0 meaning ‘rest’ and 10 meaning ‘maximal effort’. The intensity of exercise was determined by the tension of the elastic band and participants were trained to adjust the tension of the elastic band to the required level during the mid-term evaluation session. For those who had already achieved the designated level of exertion of the exercise with their body weight, they were instructed to continue the exercise without the elastic band. Frequency and the resting time of the exercise remained unchanged.

#### Exercise compliance, level of difficulty and the post‐exercise effects

**Fig. 6:**
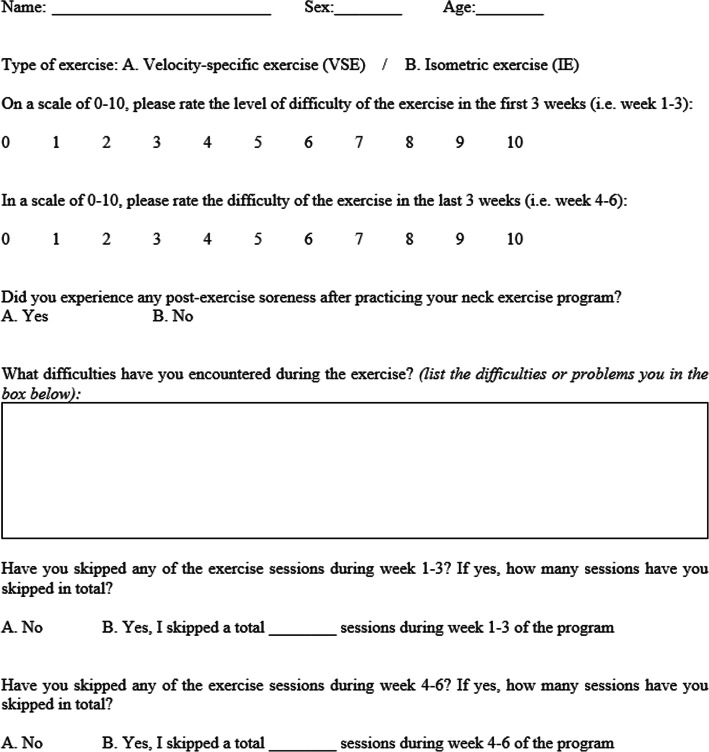
Recording of self-reported level of difficulty and post-exercise soreness, and exercise compliance

A recording sheet that consists of five questions was used to record the self-reported level of difficulty, exercise compliance and level of post-exercise soreness for all participants (Fig. 6). Comparison of these data between two exercise programs was conducted to reveal the safety and practical issues specific to the execution of exercises.

#### Statistical analysis

Intra-class correlation coefficient (ICC) was used to assess the reliability of the mean values of EMG amplitudes and torque production in flexion and extension phase, and the cross-sectional area of the deep cervical muscles. SPSS statistical analysis software (Version 23.0, IBM) was used for data analysis. Two-way repeated measure analysis variance (ANOVA) was used to examine the within and between groups difference of the outcome measures, and the group x time interaction. Pearson’s correlation coefficient was used to determine the correlation between EMG amplitude and torque production during maximal isokinetic flexion and extension for evaluation of the changes in the neuromuscular efficiency. The level of significance was set as 0.05.

## Results

The values of ICC for repeated measurements of EMG amplitude, torque and cross-sectional area of the deep cervical muscles is shown in Table 1. The ICC values range between 0.730 and 0.998 (*p* > 0.05). One of the participants from VSE group dropped out from this study because of exacerbation of neck pain after engaging in other activity. Amongst the 29 participants who completed the program, the exercise compliance levels were 82.9% and 82.6% in VSE and IE group respectively and there was no significant difference found between two groups (*p* > 0.05).

**Table 1:**
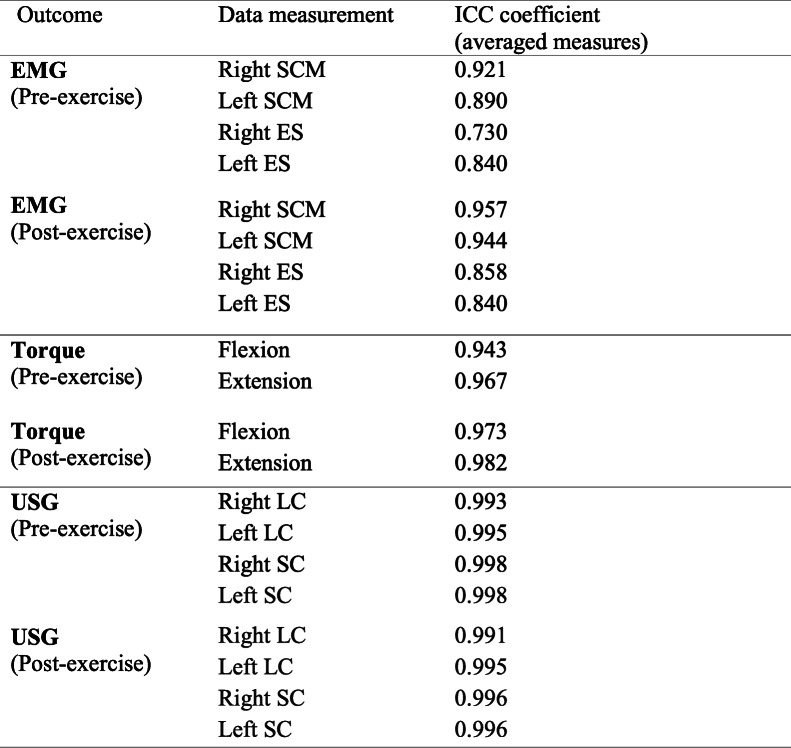
Intra-class correlation (ICC) coefficients of the outcome measures for neck muscle functions and performance, pre- and post-exercise program

### Baseline assessments

Table 2 shows the baseline data which include the demographics, baseline assessments and comparisons between VSE and IE group. There were no significant between-group differences found except for EMG amplitude of left cervical erector spinae at MVC (*p =* 0.041).

**Table 2:**
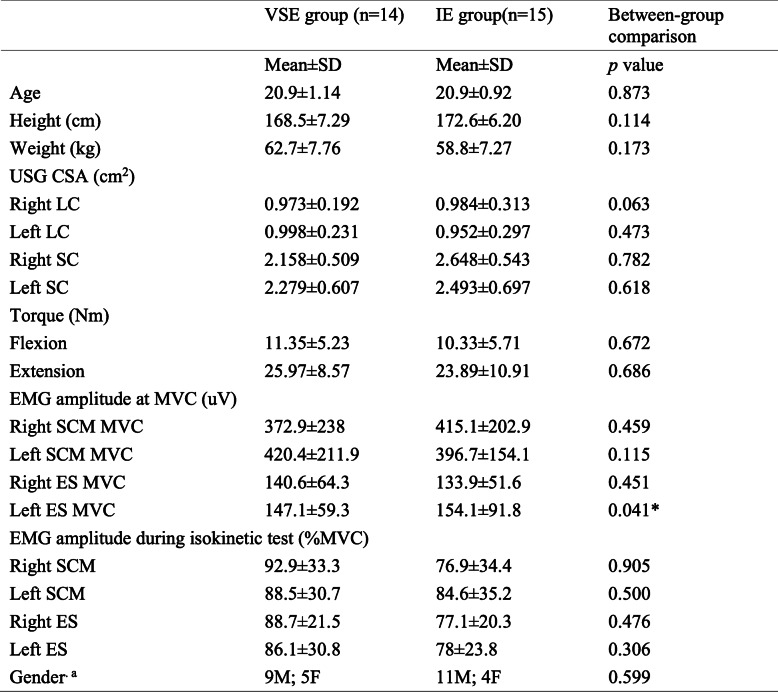
Comparison of baseline assessment between VSE and IE group

^a^ indicate Chi-Square analysis; * indicate significant between-group difference

### EMG activity of the neck flexors and extensors during isokinetic assessment

**Fig. 7:**
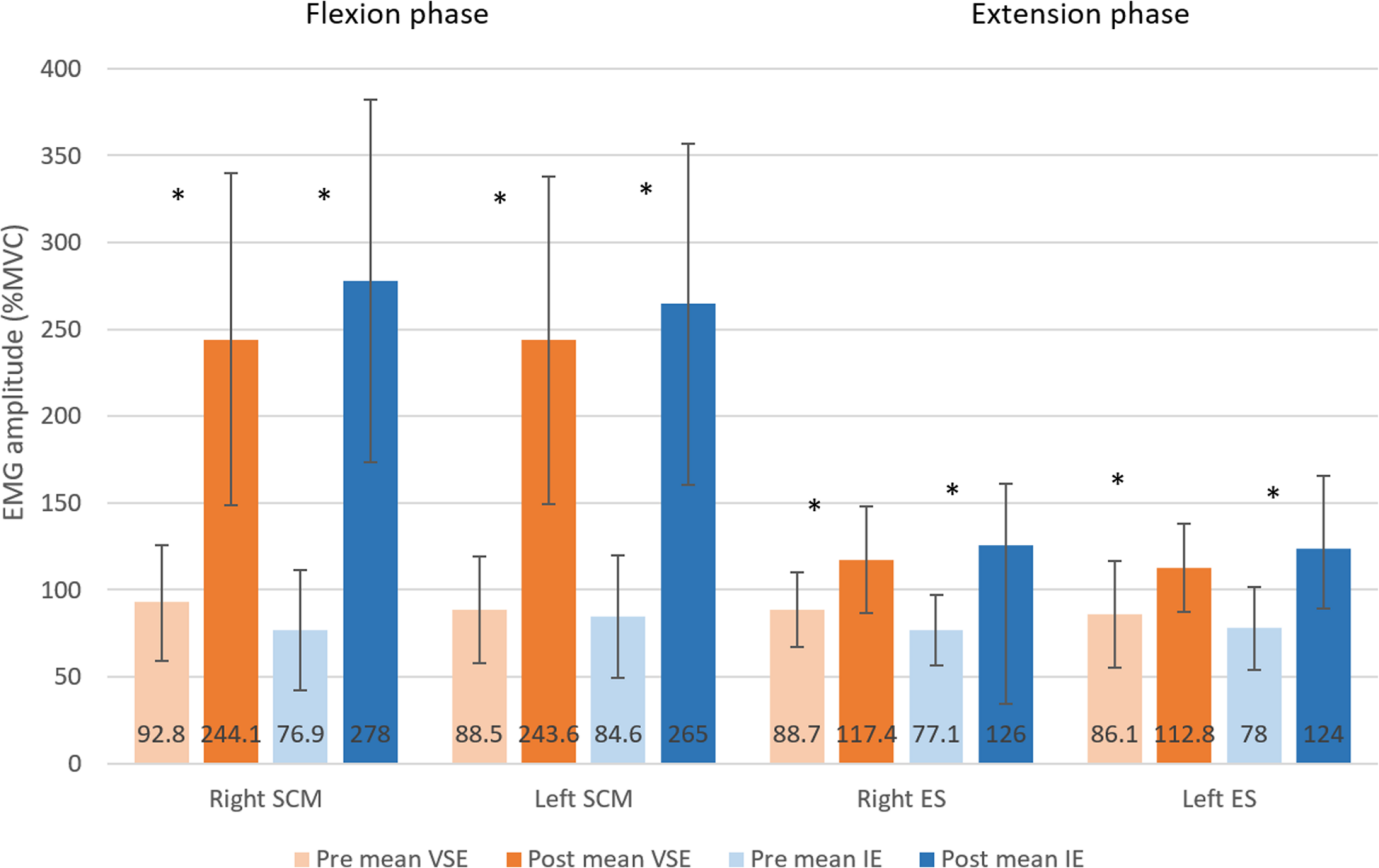
Comparisons of mean value of EMG amplitude of sternocleidomastoid during neck flexion phase and cervical erector spinae during neck extension phase of the isokinetic test between velocity-specific exercise program and isometric exercise program, pre- and post-exercise intervention. * indicates significant pre-post changes (*p*<0.05)

Figure 7 shows the EMG amplitude during isokinetic testing at pre- and post-assessment in VSE and IE group. For sternocleidomastoid and cervical erector spinae of bilateral sides, both exercise groups resulted in significant increase after the exercise program (VSE: right sternocleidomastoid: *p* < 0.001, left sternocleidomastoid: *p* < 0.001, right cervical erector spinae: *p* = 0.007, left cervical erector spinae: *p* = 0.014; IE: right sternocleidomastoid: *p* < 0.001, left sternocleidomastoid: *p* < 0.001, right cervical erector spinae: *p* < 0.001, left cervical erector spinae: *p* = 0.001). However, no significant between-group differences were found in both muscles (*p* > 0.05).

The extent of increase in EMG amplitudes of the flexors was higher than that of the extensors. In VSE group, the increase in EMG amplitudes of sternocleidomastoid (163–175% MVC) was nearly six-fold of that in cervical erector spinae (31–32% MVC). In IE group, the increase in EMG amplitudes of sternocleidomastoid (213–262% MVC) was around three-fold of that in cervical erector spinae (59–63% MVC).

### Torque performance of the neck flexors and extensors during isokinetic assessment

**Fig. 8:**
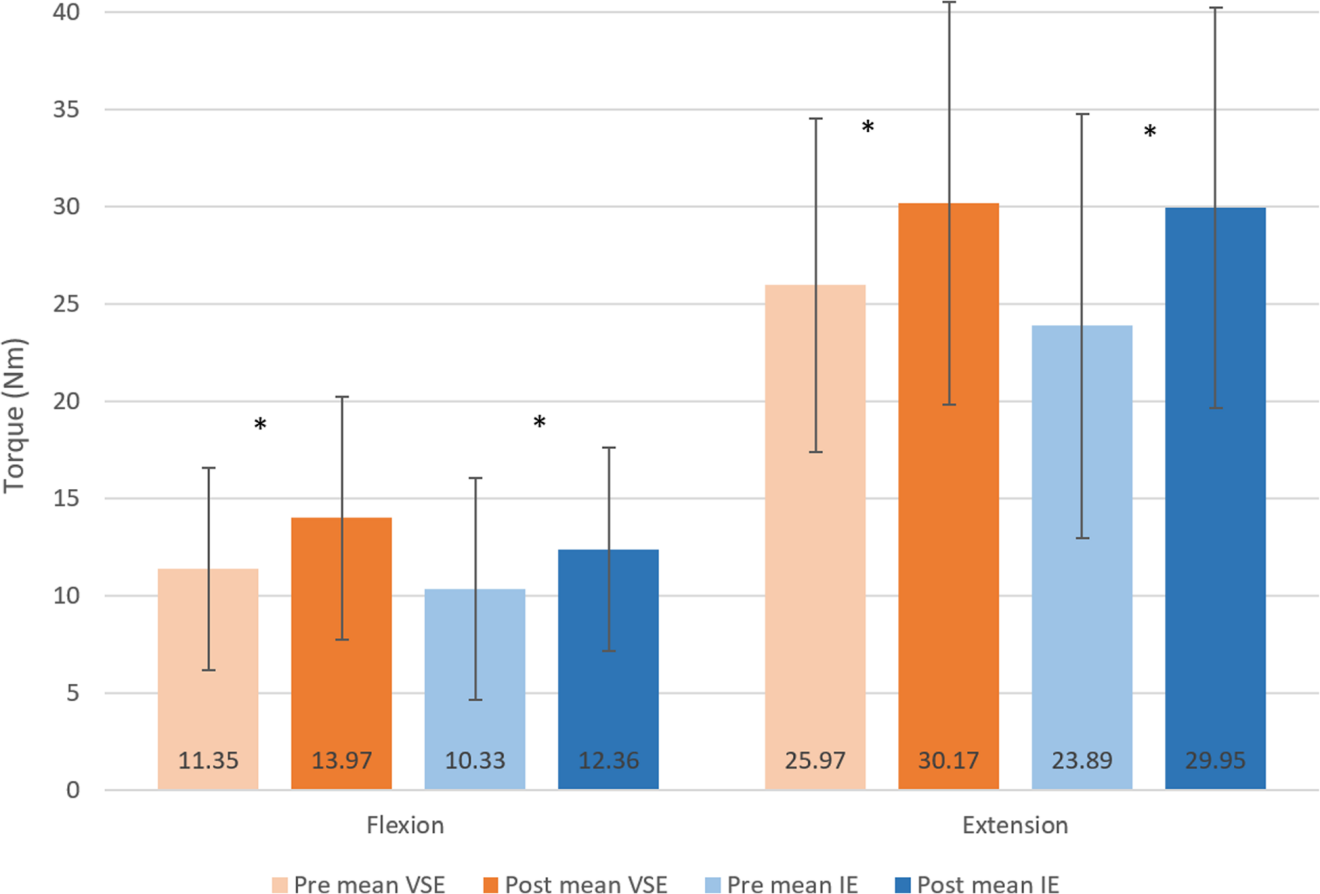
Comparisons of mean value of peak torques produced in flexion and extension phase during the isokinetic test between velocity-specific exercise group and isometric exercise group, pre- and post-exercise intervention. * indicates significant pre-post changes (*p*<0.05)

Figure 8 shows the torque performance (Nm) of flexion and extension phases at pre- and post-assessment in VSE and IE group. Both groups had significant increase in torque performance in both phases after exercise (VSE: flexion: *p* = 0.013, extension: *p* = 0.007; IE: flexion: *p* = 0.037, extension: *p* = 0.002). No significant differences were identified between groups (*p* > 0.05).

### Cross‐sectional area of the deep neck flexors and extensors

**Fig. 9:**
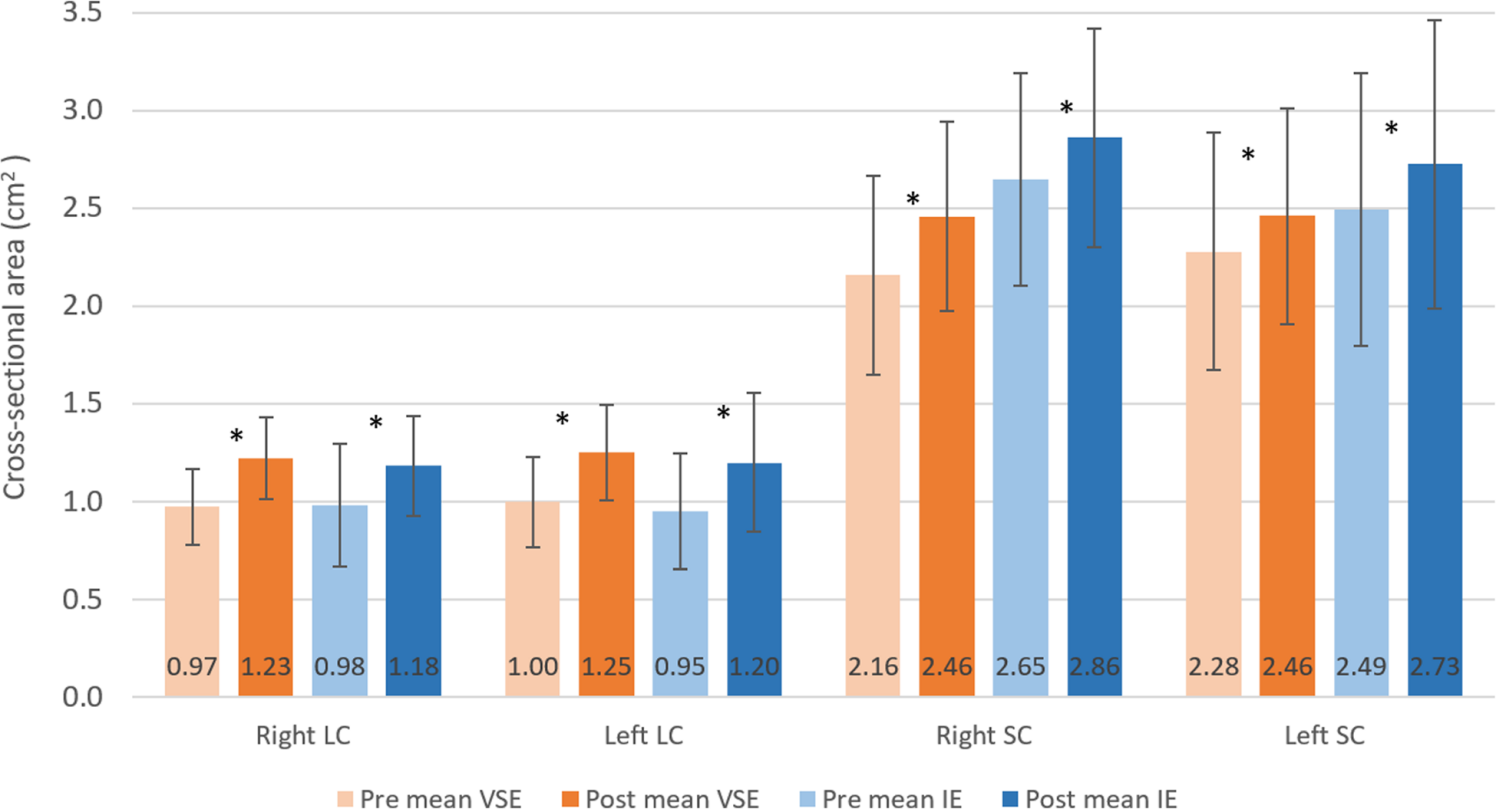
Comparisons of mean value of cross-sectional area of bilateral longus colli and semispinalis capitis (SC) between velocity-specific exercise group and isometric exercise group, pre and post exercise intervention. * indicates significant pre-post changes (*p*<0.05)

Figure 9 shows the cross-sectional area of bilateral longus colli and semispinalis capitis at pre- and post-assessment in VSE and IE group. Both exercise groups showed significant increase of muscle size in both longus colli and semispinalis capitis after exercise (VSE: right longus colli: *p* = 0.001, left longus colli: *p* < 0.001, right semispinalis capitis: *p* = 0.004, left semispinalis capitis: *p* < 0.001; IE: right longus colli: *p* < 0.001, left longus colli: *p* < 0.001, right semispinalis capitis: *p* = 0.002, left semispinalis capitis: *p* < 0.001). However, there were no significant differences between two groups.

### Correlation between EMG and torque performance of the neck flexors and extensors

**Fig. 10:**
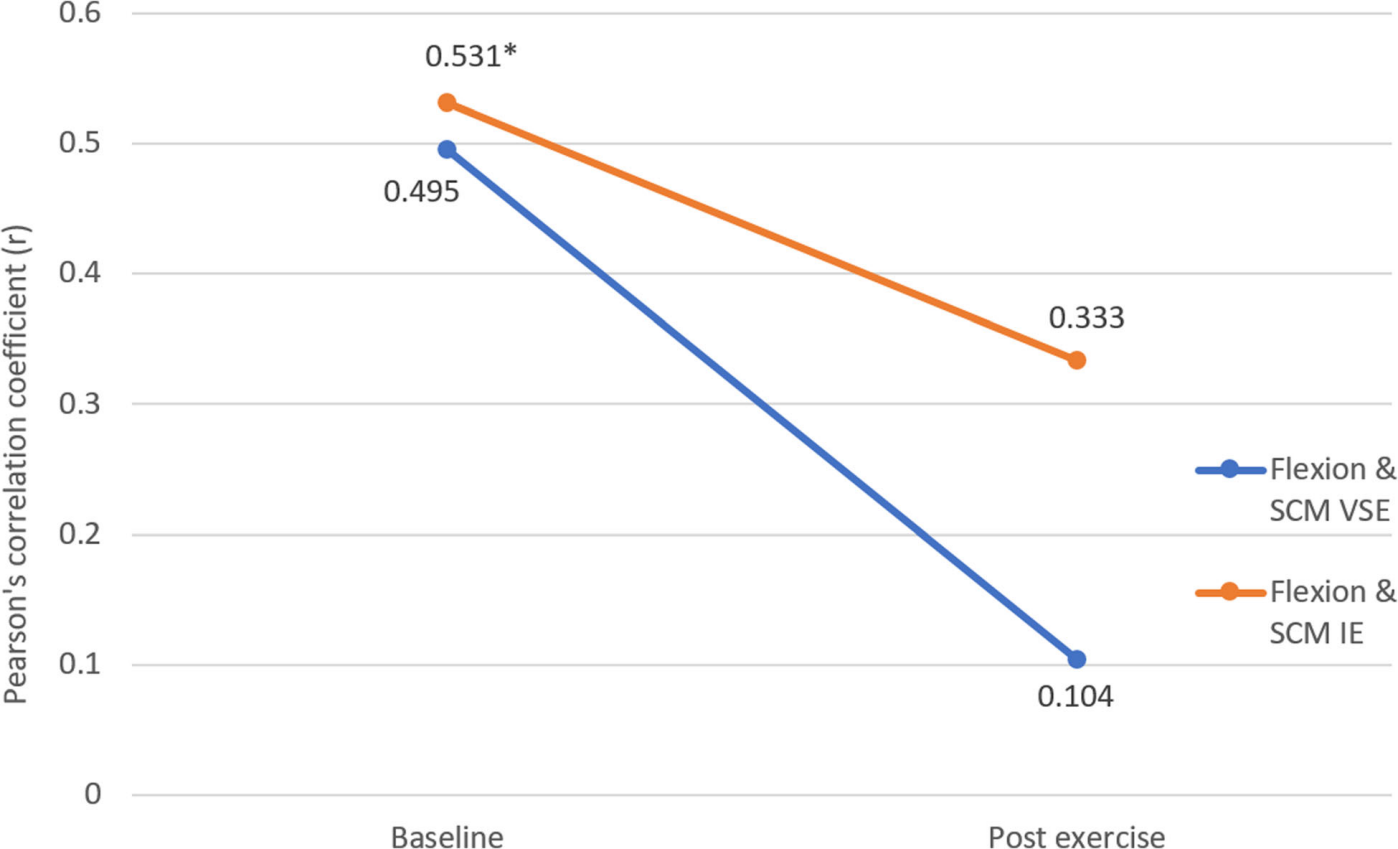
Correlation between flexion torque and sternocleidomastoid EMG amplitude in velocity-specific exercise group and isometric exercise group, pre- and post-exercise intervention. * indicates significant correlation (*p*<0.05)

Figure 10 shows the correlation between flexion torque and sternocleidomastoid EMG amplitude at pre- and post-assessment. Both groups showed a decrease in strength of correlation after exercise. Statistically significant correlation was only found in baseline measurements of the IE group (*p* = 0.041).

**Fig. 11:**
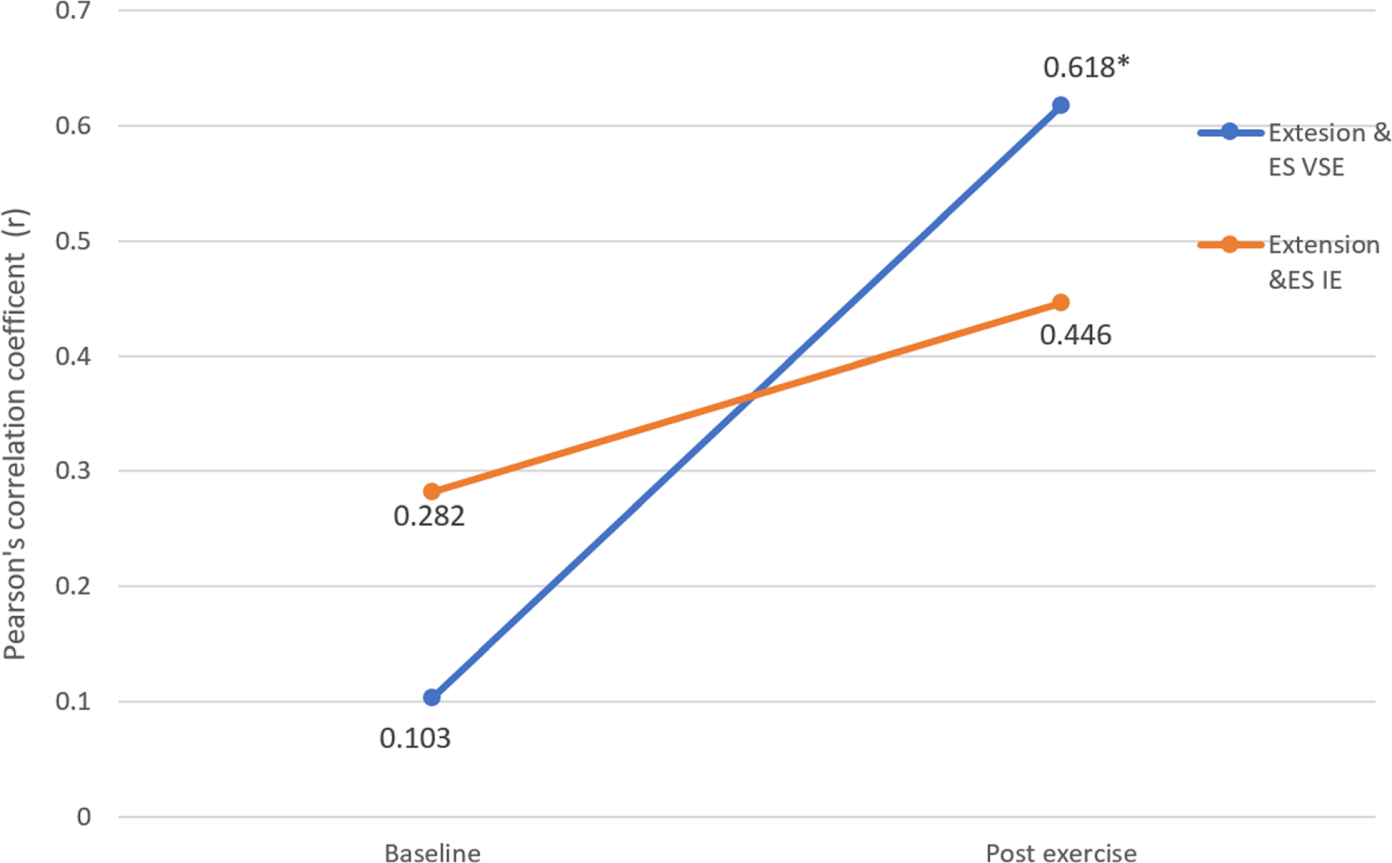
Correlation between extension torque and cervical erector spinae EMG amplitude in velocity-specific exercise group and isometric exercise group, pre- and post-exercise intervention. * indicates significant pre-post changes (*p*<0.05)

Figure 11 shows the correlation between extension torque and cervical erector spinae EMG amplitude at pre- and post-assessment. Both groups showed an increase in correlation after exercise. VSE group (*r* = 0.515) showed a greater increase in correlation compared to IE group (*r* = 0.164). Statistically significant correlation was only found in post exercise measurements of VSE group (*p* = 0.018).

### Level of difficulty, compliance of exercise programs and the post‐exercise effects

Table 3 shows the comparison of self-reported level of exercise difficulty and post-exercise soreness between VSE group and IE group. Using numeric rating scale, the VSE group and IE group rated a difficulty of 3 (range 0–6) and 3.53 (range 1–7) in the first 3 weeks. After progression of exercise was implemented by the end of 3rd week, the VSE group and IE group rated a level of difficulty of 5.64 (range 0–8) and 5.26 (range 3–7) respectively. There were no significant between-group differences on the difficulty level of the exercises at first 3 weeks and subsequent 3 weeks. Five of 14 participants (35.7%) in the VSE group and 3 of 15 participants (20%) in the IE group reported post-exercise soreness. No significant differences were observed between two groups. There were no detrimental responses or complaints post-exercise reported by the participants.

**Table 3:**
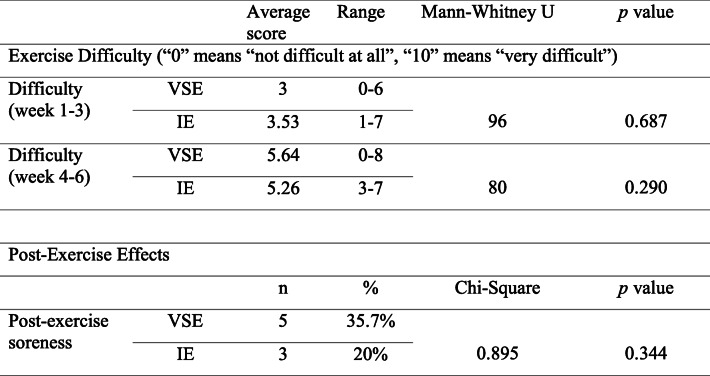
Level of difficulty and post-exercise soreness reported in VSE and IE group

## Discussion

The present results showed remarkable improvements in the muscle functions and performance in both exercise groups, substantiated by the significant increase in EMG amplitude of sternocleidomastoid and cervical erector spinae, cross-sectional area of longus colli and semispinalis capitis, and torque production. There were no statistically significant differences found between groups in terms of the magnitude of improvement.

### EMG activity during isokinetic assessment

The surface EMG amplitude of sternocleidomastoid and cervical erector spinae increased significantly in both groups after the programs. The percentage increase in sternocleidomastoid EMG amplitude was greater than that for cervical erector spinae in both groups. It remains difficult to directly compare our findings to previous studies because research studying exercise induced changes in EMG of neck muscles during isokinetic testing was limited. Previous study has reported that EMG amplitude of the cervical flexors and extensors increased linearly with increased torque production in the isometric contraction in corresponding direction [22]. The results were in agreement with the force increase found in the isokinetic muscle testing in our study. Therefore, an increase in neck surface EMG amplitude might correspond to increased force produced by the respective muscle.

### Torque performance during isokinetic assessment

The torque produced during the isokinetic testing increased in both groups, at post-assessment. The 20% and 25% increase in isokinetic strength in flexion and extension phase respectively after our 6-week IE program were comparable to the results of previous studies. A 5-week neck strengthening program for rugby players showed to have improved the isometric neck strength by 18% and 21% in flexion and extension correspondingly [23]. In addition, an 8-week lateral neck flexion IE program demonstrated a significant 9% and 20% increase in isokinetic neck strength at the fourth and eighth week respectively [24]. According to these findings, the optimal duration of IE program for neck muscle strengthening remains inconclusive due to the variations in which the exercises and evaluation of the cervical movements were conducted across studies.

Limited studies investigated the effects of VSE on the cervical spine. However, isotonic neck exercises, which shared the similar nature to VSE except for the standardised execution speed, were found to be effective for improvements in neck muscle strength. Conley et al. [25] found a 34% improvement of neck muscle strength in healthy participants when isotonic neck extension exercises were added to a 12-week general strengthening program. Mansell et al. [26] reported a 15% and 22% improvement in isometric flexion and extension strength respectively in a cohort of soccer players, after completing a 8-week isotonic neck exercise program. The extents of the improvements were comparable to the extent of increase in isokinetic strength in flexion (23%) and extension (16%) in VSE group revealed in our study. The additional strength gains from study by Conley et al. [25] might imply the necessity to evaluate the potential gains from VSE in a longer intervention period.

### Cross‐sectional area assessment of the deep neck muscles

CSA of deep neck extensors and flexors improved after exercise in both groups. For IE group, the CSA increased by 20–26% for longus colli, and 8–9% for semispinalis capitis. An 11% improvement in CSA of longus colli was found in neck pain participants who completed an 8-week neck flexion IE program [27]. The hypertrophic effect was only half of ours. With similar exercise program, it was deduced that the hypertrophic effect on longus colli might be smaller in neck pain participants. Pain might limit exercise intensity and quality of movement, reducing the strengthening effect. For deep neck extensors, our study was the first to examine the changes in CSA of semispinalis capitis by IE. The smaller hypertrophic effect on deep neck extensors with similar exercise intensity needed further investigation.

For VSE group, the CSA increased by 25–26% for longus colli, and 8–14% for semispinalis capitis. In the absence of previous data from asymptomatic group, our findings showed a two-fold increase in CSA of longus colli compared to the 12% improvement in neck pain participants after a 10-week isotonic neck flexion exercise program [28]. The differences in exercise effect on CSA of longus colli might indicate that VSE results in a more profound hypertrophy effect on deep neck flexors compared to isotonic exercise. However, such discrepancy could also be explained by the different responses towards exercise between participants with and without neck pain. For the CSA of semispinalis capitis, a 12-week isotonic neck extension exercise program showed a 24% increase in CSA of semispinalis capitis in health college students [25]. It was interesting that the muscle size continued to improve after 6th week of the program. Combined with the findings on the torque, it might be deduced that there is a potential benefit of long term VSE program for improving muscle functions and performance of the cervical spine.

The CSA of longus colli has be correlated to the neck functions and pain as previous study has showed that CSA of longus colli was smaller in individuals with neck pain [29]. The muscle size reduction might be attributed to muscle inhibition due to the changes in cervical spine motor control. On the other hand, the small muscle size could be the cause of neck pain with insufficient active stabilization. The correlation between CSA of semispinalis capitis and neck pain remained controversial. Previous study found an increased CSA of semispinalis capitis in neck pain adolescents [30]. In contrast, Fernández-De-Las-Peñas et al. [31] found a reduced deep neck extensor size in women with chronic neck pain. It should be noted that deep neck muscle size might not be the best predictor of neck pain and functions [32]. More attention should be put on neck muscle activation pattern and muscle strength instead.

### Correlation between EMG amplitude of the superficial neck flexor and extensor, and torque performance during isokinetic assessment

From the result of torque, EMG (% of MVC) and muscle CSA, there was a significant within- group difference but not between-group difference. The lack of between-group difference could possibly be related to the validity and specificity of the methods used to detect the changes in the muscle functioning related to the training. In this study, we analysed the neuromuscular control and efficiency of the muscles using the correlation between the EMG activity and functions of the corresponding muscles. While human movements are controlled by the neuromuscular system, the force production and movement are the final outputs of the neuromuscular system [33–35]. For example, given the same value of the peak torque generated in either the extension phase of the isokinetic testing, a lower EMG amplitude implies a greater torque production efficiency. However, due to the practical limitation, it was not possible to use intramuscular EMG to directly record the deep neck muscle activity level during movement testing of the present study. The explorative approach to analyze the strength of the correlation between peak torque production and EMG amplitude of corresponding muscle at respective phase of movement offers an alternative means to study the neuromuscular efficiency between the deep and superficial layer of cervical muscles. Superficial neck muscles contribute in neck movement force generation while deep neck muscles act as primary neck stabilizers [36]. However, if the stabilization role of the deep cervical muscles is compromised by the pain inhibitory mechanism and/or neuromuscular efficiency, the superficial muscles might compensate by maximizing their dual role for both neck movement force generation and segmental stabilization [6]. If there was a same peak superficial muscle EMG amplitude between two people but one needed compensation by superficial muscles, the force generation would be less effective. The association between torque production and peak EMG amplitude would become lower, implying an impaired neuromuscular efficiency.

Statistically significant correlation between the cervical erector spinae activity and neck extension torque was only found in VSE group but not in IE group. Combining the findings in investigation of the muscle size that both groups showed a similar muscle hypertrophy in semispinalis capitis, it could be deduced that VSE might be more effective in promoting the efficiency of the cervical erector spinae in torque generation and in training of deep neck extensors. Participants from VSE group might be more adapted to activate their deep neck extensors during dynamic movement while those from IE group were less accustomed due to the static nature of their exercise. Therefore, in isokinetic torque productions in IE group, semispinalis capitis were less efficient in segmental stabilization and the cervical erector spinae had to contribute in stabilization. The torque producing efficiency of the cervical erector spinae had been reduced as a result which was shown by the less significant correlation.

In contrary, correlations between sternocleidomastoid activity and neck flexion torque post-intervention were insignificant in both groups, despite the significant muscle size increase of longus colli in both groups. This manifestation could possibly be explained by the dramatic increase in sternocleidomastoid activities after intervention. A nearly three-fold increase was found in sternocleidomastoid activity, which was substantially higher than that in cervical erector spinae (around 15%). On the other hand, the muscle size of longus colli only showed a 25% increase. The extent of increase in longus colli strength might have failed to catch up with the extremely high demand for postural stability related to the increased sternocleidomastoid activity and strength. Sternocleidomastoid might have contributed to postural stability as a compensation strategy and therefore, leading to a reduced efficiency in the torque generation. By reviewing our exercise program design, it was suspected that the exercises for neck flexion might be too demanding though all participants were able to perform the exercise safely. The increase in CSA of longus colli (20–26%) was two-fold of that in semispinalis capitis (8–14%). It might imply that the flexion exercise intensity was much higher than that of extension in both groups. With participants being instructed to maintain chin tucked-in during flexion exercise, a greater demand of the longus colli excursion is postulated in addition to the load caused by the gravity and the elastic band. As a results, some participants might have adopted different muscle activation patterns during the exercise implementation.

#### Safety and comfort of the exercises

All the participants performed the exercises safely. Attempts were made to protect the participant’s neck. Participants were required to keep their chin tucked-into reduce shearing force on the cervical spine. No detrimental responses or complaints about difficulty were reported by the participants. Difficulty of the exercises was reported from 0 to 8/10. There were only a few reports of temporary post-exercises soreness from both groups. Chiu et al. revealed that no adverse events were reported by participants with neck pain when performing the isotonic neck exercise program [37]. These indicate that VSE would be generally safe for training of the cervical spinal muscles.

#### Limitations

There were several limitations in the study. The sample size was small with only 29 participants which may contribute to the reduced statistical power. Besides, our exercise program duration was relatively short. A previous study had shown that isometric exercise had strengthening effect on muscle even after 3 years [38], therefore, the long-term effect of VSE is yet to be investigated. With only the asymptomatic participants being recruited in this study, future studies are required to investigate the effects of VSE in population with neck pain [6].

Participants recruited in this study were relatively young and 62.1% of them exercised regularly. This might explain the greater size of deep neck muscle revealed at baseline compared to previous studies. The mean values of CSA of bilateral longus colli of our participants were also slightly greater (0.97 cm^2^ compared to 0.85–0.87 cm^2^). The differences are even greater for semispinalis capitis (2.39–2.41 cm^2^ compared to 1.70–1.73 cm^2^) [39, 40]. In view of the heterogeneities of participants between studies, our findings would not be generalizable to older and more sedentary populations.

#### Clinical implication and future study

Findings of the present study showed that VSE were comparable with IE in improving neck functions and performance. VSE might be superior to IE in promoting efficiency of superficial neck muscle in torque generation. It had been shown by various studies that IE were effective in improving neck functions and reducing neck pain intensity, and hence they were prescribed to participants with neck pain [13, 41]. With the findings from this study, VSE might serve as an alternative to IE in neck training and rehabilitation. Future studies are recommended to investigate the immediate and long-term effects of VSE on recovery of patients with neck dysfunction. In addition, other parameters, for example, the composition and type of muscle fiber of neck should be examined to better reveal the physiological mechanisms underlying the changes induced by VSE in neck pain participants and substantiate its clinical efficiency.

## Conclusions

This study showed that both velocity-specific exercise and isometric exercise resulted in a comparable degree of improvement in functions and performance of the cervical flexors and extensors. Our finding confirms that velocity-specific exercise can enhance the neuromuscular efficiency of cervical extension in healthy individuals. It is suggested that velocity-specific exercise can be a safe alternative to training and rehabilitation of the cervical spine. Further studies are recommended to investigate the clinical efficacy of this exercise for people with neck pain.

## Data Availability

The datasets supporting the conclusions of this article are included within the article. The raw data can be requested from the corresponding author.

## Abbreviations

ANOVA: Analysis of variance
BPM: Beats per minute
CSA: Cross-sectional area
EMG: Electromyography
ICC: Intra-class Correlation Coefficient
IE: Isometric exercise
MVC: Maximal voluntary contraction
SENIAM: Surface Electromyography for Non-invasive Assessment of Muscles
VSE: Velocity-specific exercise
